# Neural mechanisms of dopamine function in learning and memory in *Caenorhabditis elegans*

**DOI:** 10.1042/NS20230057

**Published:** 2024-01-18

**Authors:** Anna McMillen, Yee Lian Chew

**Affiliations:** College of Medicine and Public Health and Flinders Health and Medical Research Institute, Flinders University, Bedford Park, 5042, South Australia, Australia

**Keywords:** Caenorhabditis elegans, dopamine, G-protein-coupled receptors, learning and memory

## Abstract

Research into learning and memory over the past decades has revealed key neurotransmitters that regulate these processes, many of which are evolutionarily conserved across diverse species. The monoamine neurotransmitter dopamine is one example of this, with countless studies demonstrating its importance in regulating behavioural plasticity. However, dopaminergic neural networks in the mammalian brain consist of hundreds or thousands of neurons, and thus cannot be studied at the level of single neurons acting within defined neural circuits. The nematode *Caenorhabditis elegans (C. elegans)* has an experimentally tractable nervous system with a completely characterized synaptic connectome. This makes it an advantageous system to undertake mechanistic studies into how dopamine encodes lasting yet flexible behavioural plasticity in the nervous system. In this review, we synthesize the research to date exploring the importance of dopaminergic signalling in learning, memory formation, and forgetting, focusing on research in *C. elegans*. We also explore the potential for dopamine-specific fluorescent biosensors in *C. elegans* to visualize dopaminergic neural circuits during learning and memory formation in real-time. We propose that the use of these sensors in *C. elegans*, in combination with optogenetic and other light-based approaches, will further illuminate the detailed spatiotemporal requirements for encoding behavioural plasticity in an accessible experimental system. Understanding the key molecules and circuit mechanisms that regulate learning and forgetting in more compact invertebrate nervous systems may reveal new druggable targets for enhancing memory storage and delaying memory loss in bigger brains.

## Introduction

Dopamine plays an important role in the nervous system of many organisms, with multiple conserved functions. Dopamine deficiency in diverse species results in impaired movement, fewer regulated responses to incentives involved with survival, and interruptions to learning and memory formation [1,2]. Human conditions in which these behaviours are affected include Parkinson’s disease, schizophrenia, attention-deficit/hyperactivity disorder (ADHD), depression, and addiction, all of which have been linked with dopamine deficiency [3–8]. Although a great deal of mechanistic insight into how dopamine regulates learning and memory has been gained using mammalian models, mammals have a large and highly complex nervous system that hinders the ability to study dopaminergic circuits at the level of single neurons, something that is possible with *Caenorhabditis elegans (C. elegans)* as the connectome of their entire nervous system, consisting of 300 neurons, has been fully mapped [9,10]. This, in addition to the well-characterized genome and genetic tractability of the worm, makes *C. elegans* an excellent candidate for experimental examination and manipulation of neural interactions that take place during memory formation. Although *C. elegans* is an invertebrate and has a relatively small and compact brain, several important aspects of learning and memory are conserved between worms and higher organisms: worms display habituation and sensitization (non-associative learning) as well as classical conditioning (associative learning) and express both short- and long-term memories. In addition, several genetic regulators of learning in mammals are also required in *C. elegans*, including cAMP-response element-binding protein (CREB) [11] calcium/calmodulin-dependent protein kinase II (CaMKII) [12] and AMPA receptor subunit GLUR1 [13,14].

In this review, discoveries pertaining to the role of dopamine in learning, memory and forgetting in *C. elegans* will be detailed. Although other organisms will be mentioned in their relevance to the overall state of research on dopamine, and where discoveries of the role of dopamine in other organisms suggests there is a gap in research in *C. elegans*, they will not be examined in detail here. Many other reviews detail what is known about the role of dopamine in learning in mammals, including [15–17]. In addition, the transparent nature of *C. elegans* facilitates the use of fluorescent techniques to interrogate the role of neurotransmitters like dopamine in driving key brain functions, such as using genetically encoded calcium indicators (GECIs) to visualize neural activity or fluorescent biosensors to visualize specific neurons, neurotransmitter-receptor binding, and signal transduction [18–20]. We will discuss current techniques for real-time visualization of dopaminergic circuits in living animals, and how this will provide new mechanistic insight into how dopamine, and potentially other neuromodulators, function to regulate plasticity in the nervous system.

## Dopaminergic neural circuits in *C. elegans*

**Figure 1:**
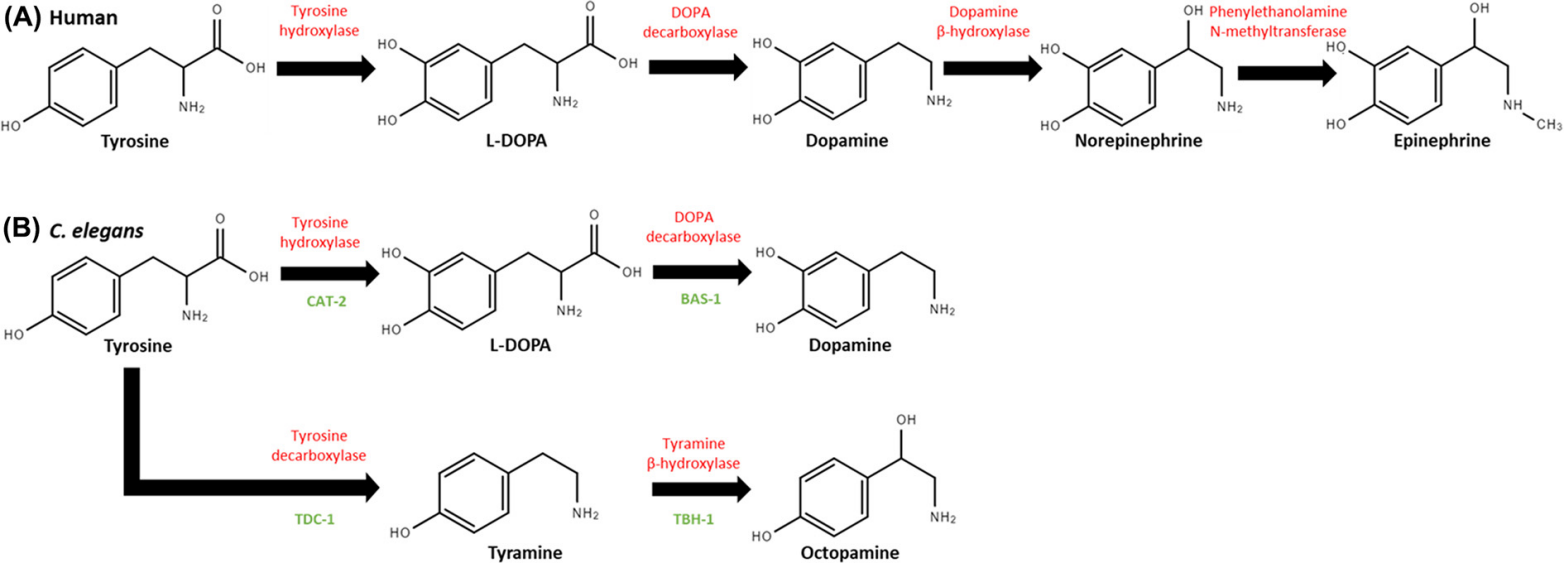
Catecholamine family synthesis pathways from amino acid tyrosine Enzymes are labeled in red. (**A**) shows the catecholamine synthesis pathway from tyrosine in humans. The enzyme tyrosine hydroxylase converts tyrosine to L-dihydroxy-phenylalanine (L-DOPA) by adding a hydroxyl group. This is the rate-limiting step for the synthesis of both dopamine and norepinephrine synthesis. A L-DOPA decarboxylase enzyme removes the carboxyl group from L-DOPA to make dopamine. Dopamine β-hydroxylase adds a hydroxyl group to dopamine to make norepinephrine. A methyl group is added to norepinephrine by the enzyme phenylethanolamine-N-methyltranferase. (**B**) shows the catecholamine synthesis pathway from tyrosine in C. elegans. The gene names for the encoded enzymes for each reaction are written in green. Like in (**A**), a tyrosine hydroxylase adds a hydroxyl group to tyrosine to make L-DOPA. This is still the rate-limiting step for dopamine synthesis. In C. elegans the gene that encodes for this tyrosine hydroxylase is cat-2. Also like in (**A**), a DOPA decarboxylase removes a carboxyl group to make dopamine. Worms do not make norepinephrine or epinephrine. In a secondary pathway in C. elegans, tyrosine can also synthesize tyramine and octopamine. A tyrosine decarboxylase removes the carboxyl group from tyrosine to make tyramine. A tyramine β-hydroxylase then adds a hydroxyl group to tyramine to get octopamine. The tyrosine hydroxylase that makes L-DOPA and the tyramine β-hydroxylase that makes octopamine require a GTP cyclohydroxylase 1 cofactor that is encoded by cat-4 [1,21,28,29].

For the purposes of this review, we define neurotransmitters as chemicals that signal across the synaptic cleft in a ‘point-to-point’ manner, affecting only a limited number of post-synaptic cells after release [21–23], while neuromodulators act in a diffuse manner on groups of neurons, producing longer-range and/or longer-lasting effects, often through the initiation of molecular signalling cascades or activating second messengers [21,24]. We note that a clear distinction between a neurotransmitter and a neuromodulator is difficult as (i) although most neuromodulators are thought to act through G protein-coupled receptors (GPCRs), neurotransmitters are known to act through both ionotropic and metabotropic (G protein-coupled) receptors, and (ii) chemicals that function both in a point-to-point and diffuse manner i.e. as both neurotransmitters and neuromodulators, may act through ionotropic receptors when released in a diffuse manner [25,26]. According to this definition, dopamine can act as both a neurotransmitter and neuromodulator, and is not limited to rapid effects on directly surrounding neurons. Dopamine is a catecholamine derived from the essential amino acid tyrosine, along with epinephrine, norepinephrine, tyramine, and octopamine [21,27–29]. Figure 1 shows the similarities and differences in the catecholamine synthesis pathway between humans and *C. elegans*; showing that in worms, tyramine and octopamine are formed in an alternative pathway instead of norepinephrine and epinephrine in humans. This alternative pathway requires tyrosine decarboxylase (*tdc-1* in worms) to make tyramine from tyrosine and tyramine β-hydroxylase (*tbh-1* in worms) to make octopamine from tyramine. In both species, the rate-limiting steps to dopamine synthesis remain conserved.

In *C. elegans*, CAT-2 encodes for a tyrosine hydroxylase that is the forementioned rate-limiting step required to synthesize L-DOPA from tyrosine (Figure 1) [30]. Interestingly, *cat-2* mutant worms have approximately a 40% reduction in dopamine but do not eliminate dopamine entirely [22]. A similar observation was made in a study on tyrosine hydroxylase-deficient mice, which showed that catecholamines can also be synthesized through an alternative pathway requiring tyrosinase, which converts tyrosine to L-Dopa during melanin synthesis [31]. There are six tyrosinase family members in *C. elegans* – the most well-studied member *tyr-2* was shown not to have any tyrosinase activity [31], although other family members have not been tested. It is therefore possible that there may be other methods of dopamine synthesis in *C. elegans*, as in mice.

### Dopaminergic neurons in *C. elegans*

**Figure 2:**
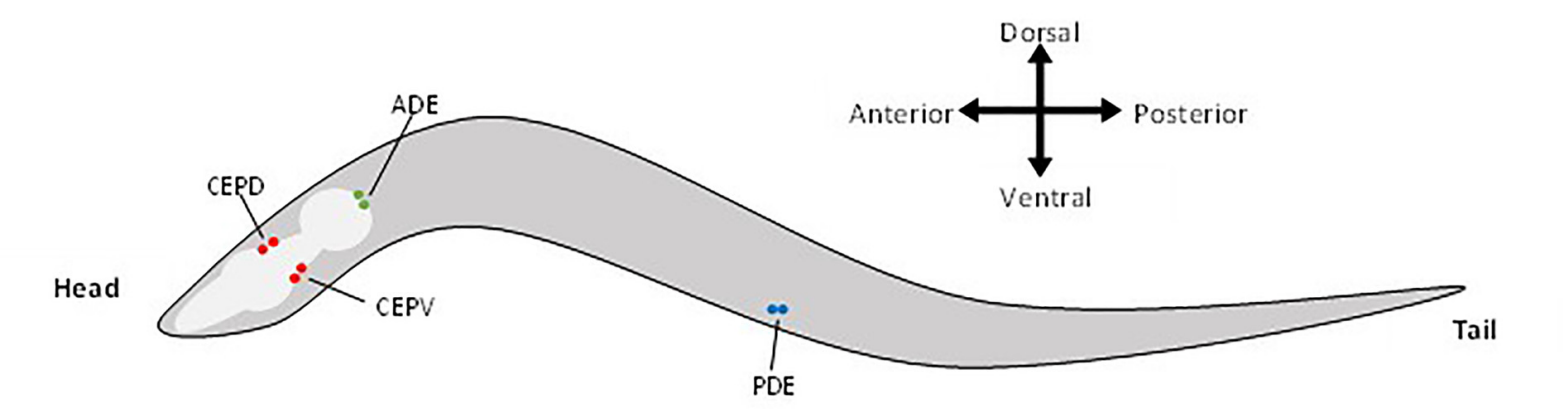
Schematic of hermaphrodite *C. elegans* with labeled dopaminergic neurons There are four cephalic sensilla (CEP) neurons, two anterior deirids (ADE) neurons, and two posterior deirids (PDE) neurons. The CEP neurons are located behind the first bulb of the pharynx and extending to the tip of the nose. One pair of CEP neurons are located on the dorsal side (CEPDL and CEPDR) and the other on the ventral side (CEPVL and CEPVR) of the pharynx. The pair of ADE neurons (ADEL and ADER) are located lateral to the second bulb of the pharynx. The pair of PDE neurons (PDEL and PDER) are in the posterior half of the worm body. Red indicates CEP neurons, green indicates ADE neurons, and blue indicates PDE neurons. The light gray is the worm’s pharynx. In the worm anterior is towards the head, posterior is towards the tail, dorsal is the top side of the worm, and ventral is the bottom side that the worm moves on [10,23,24].

Of the 300 neurons in hermaphrodite *C. elegans*, only 8 synthesize dopamine, with male worms having an additional 6 dopaminergic neurons in the tail [23,24]. As shown in Figure 2, the 8 sex-shared dopaminergic neurons are a pair of mechanosensory neurons located in the head known as ADE, two pairs of mechanosensory neurons located in the head and extending to the nose called the CEP neurons, and a pair of neurons with ciliated receptor endings in the posterior of the worms known as PDE [10]. ADE, PDE, and CEP neurons have all been implicated in modulating area-restricted searching, slowing locomotion when on a food patch, and in specific mechanosensory responses [22,32,33]. Other locomotion phenotypes attributed to these dopaminergic neurons include regulating body bend frequency (requiring the TRP channel *trp-4*) [34] and the ‘swim’ to ‘crawl’ transition upon moving from liquid to solid media [35]. In addition, both CEP and ADE neurons are important in learned bacteria avoidance behaviour and mitochondrial stress-induced aversive learning [36].

*C. elegans* are highly amenable to molecular tools such as neural ablation or genetic knock-down, which facilitates the discovery of which dopaminergic cells or receptors are required for specific behaviours [24,33,37–39]. In the following sections, we summarize the key components of dopamine signalling in *C. elegans*, as well as the aspects of learning and memory that are modulated by dopaminergic neural circuits. It is important to note that although multiple aspects of dopamine synthesis and signalling signaling pathways are conserved between *C. elegans* and other organisms, dopaminergic neural circuits do differ between species [40]. For example, in *C. elegans* there are few dopaminergic neurons that primarily act in an ‘extra-synaptic’ manner on over 100 neuronal targets (based on receptor expression), predominantly motor neurons [41]. This is different from mammals, in which dopaminergic neurons are mainly concentrated in nuclei in the ventral midbrain – the substantia nigra pars compacta (SNc) and the ventral tegmental area (VTA), which in turn project onto distinct regions in the basal ganglia, amygdala and prefrontal cortex (see [42,43] for review). Although dopaminergic neural circuits differ in architecture and makeup between worms and mammals, it is clear that a role for dopamine in learning and memory is conserved between species.

### Dopamine receptors and reuptake in *C. elegans*

Like other neurotransmitters, dopamine receptors in the worm can be broadly divided into two categories, ligand-gated ion channels (LGIC) and G protein-coupled receptors (GPCRs). LGICs have a pore within the plasma membrane that is closed until a specific neurotransmitter binds to the receptor, opening the channel to allow select ions to move across the plasma membrane [21,44]. LGICs open and desensitize quicky, ideal for short-term signalling between neurons [44,45]. LGC-53 is an example of a dopamine-gated chloride channel in *C. elegans* [46].

In contrast, GPCRs are not ionotropic receptors and do not directly impact the membrane potential in the same manner as LGICs, but instead produce cell signalling through the activation of signal transduction pathways, a slower process that produces more long-lasting effects [21,45]. GPCRs, also known as seven transmembrane receptors, have a single polypeptide that crosses the cellular membrane seven times [47]. Dopamine binds to the receptor on the extracellular side of the membrane and causes a conformational change, the largest change occurring between the 5th and 6th transmembrane domains [21,47–49]. This causes the activation of G-protein signalling cascades. Dopaminergic GPCRs are broadly divided into D1-like and D2-like categories, named for the different roles dopamine was found to play via mammalian D1 and D2 receptors; these roles have been found to be conserved in dopamine receptors in diverse organisms [1,2,47]. D1-like receptors couple with stimulatory G-proteins to stimulate adenylyl cyclase and raise the amount of cAMP, thus stimulating the cAMP signalling pathway [2]. Conversely, D2-like receptors couple with inhibitory G-proteins when a dopamine molecule is bound to the receptor and thus inhibit adenylyl cyclase, decreasing cAMP levels. In *C. elegans* DOP-1 has been found to be a D1-like receptor while DOP-2 and DOP-3 are both D2-like receptors [1,47]. Other GPCRs DOP-5 and DOP-6 are believed to bind dopamine and act as dopamine receptors, but this has not yet been shown biochemically [41]. DOP-4 in *C. elegans* is neither D1 or D2-like but instead an invertebrate-specific dopamine receptor [1].

Outside of learning and memory, detailed below, dopamine receptors have been shown to play a significant role in the worm’s ability to distinguish changes in their environment [50,51]. *C. elegans* can determine spatial differences in their environment that requires the DOP-3 receptor and mechanosensory TRP-4 channel [51]. Worms also use a combination of mechanosensation and proprioception senses to develop response strategies to navigate a choice maze that is dependent on DOP-3 and the *C.elegans* CREB homolog [50].

Dopamine that does not traverse the synaptic cleft and bind to receptors can remain in the synaptic cleft until it is cleared or the cells reuptake the chemical. DAT-1 encodes a dopamine membrane transporter that is a sodium-dependent symporter, allowing dopamine re-uptake from the synaptic cleft [33,52]. While worms with a null mutation in this gene can still synthesise dopamine, the amount of residual dopamine in the nervous system is limited. Indeed, too much dopamine in the synaptic cleft can result in swimming induced paralysis in the worm, where reuptake of dopamine by DAT-1 is required to prevent this paralysis [53]. Using *cat-2* and *dat-1* mutants to study dopaminergic signalling has been broadly used to examine the role of dopamine in regulating *C. elegans* behaviours. We have summarized the role of important genes involved in dopaminergic signalling in *C. elegans*, including dopamine receptors and rate-limiting synthesis enzymes in Table 1.

**Table 1:** Important genes involved in dopamine signalling, the impacted behaviours, and the role they play in learning and memory in *C. elegans*

Gene	Function	Expression patterns	Behaviours impacted by mutants	Role in learning and memory
*dop-1*	A D1-like GPCR that has an antagonistic relationship with *dop-3* [1,22,47,53,119].	Expresses in neurons in the head, cholinergic motor neurons in the ventral cord, and in the tail [119].	Model for worms that have functioning dopamine signalling except for *dop-1* receptors [119].	Necessary for learned bacterial avoidance under mitochondrial stress [36].
		Expresses in mechanosensory neurons PVD [119].	Double mutant with *dop-2* less able to learn bacterial avoidance under mitochondrial stress [36].	Involved in mechanosensory habituation [22].
			Habituates to a mechanosensory stimulus faster than wild-type worms [22].	Necessary for state-dependent mechanosensory habituation with food [33].
			Habituates to a mechanosensory stimulus faster than wild-type worms when on food, at a similar rate to wild-type worms off food [33].	Necessary for the transition in movement states between swimming and crawling when changing state of environment [120].
			Impaired movement when exiting a puddle [120].	Involved in the increased probability of off lawn egg laying in response to predators [86].
			Slightly decreased probability of off lawn egg laying in response to predators [86].	
*dop-2*	A D2-like GPCR auto-receptor that can create a negative feedback loop for dopamine release in response to dopamine in the synaptic cleft [1,47,121–123].	Expresses in all dopaminergic neurons (including the ray neurons in the tail male worms), some neurons around the nerve ring, and some neurons on the posterior side of the body [123].	Model for worms that have functioning dopamine signalling except for *dop-2* receptors [38].	Necessary for learned bacterial avoidance under mitochondrial stress [36].
			Double mutant with *dop-1* less able to learn bacterial avoidance under mitochondrial stress [36].	Involved in mechanosensory habituation [47].
			Habituates to a mechanosensory stimulus faster than wild-type worms [47].	Involved in the increased probability of off lawn egg laying in response to predators [86].
			Slightly decreased probability of off lawn egg laying in response to predators [86].	
*dop-3*	A D2-like GPCR that has an antagonistic relationship with *dop-1* [1,47,53,119].	Expresses in neurons in the head, strongly in the GABAergic motor neurons in the ventral cord, weakly in the cholinergic motor neurons in the ventral cord, weakly in some body-wall muscles, and in the tail [119].	Model for worms that have functioning dopamine signalling except for *dop-3* receptors [119].	Necessary for learned bacterial avoidance under mitochondrial stress [36].
		Expresses in mechanosensory neurons PVD [119].	Less sensitive to locomotion paralysis by exogenous dopamine [119].	Modulates state-dependent preference for environmental density with food [51].
			Does not perform basal slowing response when encountering food [119].	Involved in learning bacteria avoidance when paired with 2-nonanone [73].
			Induces swimming-induced paralysis when suspended in liquid environments [53].	Necessary for learning the location of food in a maze and recalling based on proprioception combined with mechanosensation [50].
			Does not have a decreased movement frequency in simulated zero gravity conditions [124].	Involved in the increased probability of off lawn egg laying in response to predators [86].
			Displays less preference for density of the environment [51].	
			Less able to learn bacterial avoidance under mitochondrial stress [36].	
			Does not show increased avoidance behaviour to 2-nonanone [73].	
			Reduced ability to locate food in a maze or choose maze arm where food was previously after conditioning [50].	
			Slightly decreased probability of off lawn egg laying in response to predators [86].	
*dop-4*	An invertebrate-specific dopamine GPCR [71,119].	Expressed in neurons I1, I2, ASG, AVL, CAN, and PQR, in the vulva, the intestine, the rectal glands, rectal epithelial cells, weak expression in some neurons in the head, and in ray 8 in males [125].	Model for worms that have functioning dopamine signalling except for *dop-4* receptors [1,71].	Necessary for state-dependent modulation of copper II chloride avoidance with food [71].
			Does not show enhanced avoidance to copper II chloride when food is present [71].	Necessary for the transition in movement states between swimming and crawling when changing state of environment [120]
			Impaired movement when exiting a puddle [120].	
*lgc-53*	A dopamine-gated chloride channel [46].	Expressed in neurons IL2, URY, AIM, FLP, AVF, HSN, PVD, VA, and sometimes CAN [41].	Model for worms that have functioning dopamine signalling except for *lgc-53* receptors [46].	No information at this time
*cat-1*	A transporter that loads neurotransmitters into vesicles and enables symporter activity for dopamine-sodium and serotonin-sodium [1,84,126].	Expressed in all dopaminergic neurons, all serotonergic neurons, male neurons, and the neurons RIC and CAN [53,127].	Model of worms that are simultaneously dopamine and serotonin deficient by being unable to pack these neurotransmitters into vesicles [126].	Modulates ethanol state-dependent learning of benzaldehyde adaptation [84].
			Does not require ethanol present during testing if ethanol was present during learning [84].	Involved in the increased probability of off lawn egg laying in response to predators [86].
			Decreased probability of off lawn egg laying in response to predators [86].	
*cat-2*	A tyrosine hydroxylase necessary to synthesize dopamine [30].	Expresses in all dopaminergic neurons (including the ray neurons in the tail male worms) [30].	Model of worms that are dopamine deficient by being unable to synthesize dopamine, although they retain 30-40% of dopamine [22,30].	Modulates ethanol state-dependent learning of benzaldehyde adaptation [84].
			Displays less preference for density of the environment [51].	Necessary for learned bacterial avoidance under mitochondrial stress [36].
			Does not perform area-restricted searching behaviour to look for food [32].	Modulates state-dependent preference for environmental density with food [51].
			Does not perform basal slowing response when encountering food [119,128].	Involved in mechanosensory habituation [22].
			Does not require ethanol present during testing if ethanol was present during learning [84].	Necessary for state-dependent mechanosensory habituation with food [33].
			Less able to learn bacterial avoidance under mitochondrial stress [36].	Necessary for state-dependent modulation of copper II chloride avoidance with food [71].
			Habituates to a mechanosensory stimulus faster than wild-type worms [22].	Necessary for appetitive associative learning of butanone and liquid food and short-term memory formation of the learned behaviour [78].
			Habituates to a mechanosensory stimulus faster than wild-type worms when on food, at a similar rate to wild-type worms off food [33].	Involved in the increased probability of off lawn egg laying in response to predators [86].
			Does not show enhanced avoidance to copper II chloride when food is present [71].	
			Reduced chemotaxis index to butanone paired with liquid food after conditioning and reduced recall at later time points [78].	
			Decreased probability of off lawn egg laying in response to predators [86].	
*cat-4*	A GTP cyclohydroxylase 1 that is a co-factor used by hydrolases to synthesize serotonin, dopamine, and octopamine [1,126].	Expressed in all dopaminergic and serotonergic neurons, in the epidermis, weakly in some anterior intestinal cells, and in some rectal epithelial cells [129].	Model for worms that are simultaneously dopamine, serotonin, and octopamine deficient by being unable to synthesize these neurotransmitters [126].	Necessary for state-dependent mechanosensory habituation with food [33].
			Does not perform basal slowing response when encountering food [128].	
			Habituates to a mechanosensory stimulus faster than wild-type worms when on food, at a similar rate to wild-type worms off food [33].	
*dat-1*	A sodium-dependent dopamine membrane transporter that clears dopamine and allows re-uptake from the synaptic cleft [33,52].	Expressed in all dopaminergic neurons (including the ray neurons in the tail male worms) [53,130].	Model of dopamine deficient worms by being unable to clear dopamine from the synaptic cleft or re-uptake dopamine to reuse [33,52].	Necessary for learned bacterial avoidance under mitochondrial stress [36].
	Inhibits the build-up of endogenous dopamine to not impact extrasynaptic sites [53].		Induces swimming-induced paralysis when suspended in liquid environments [53].	Necessary for state-dependent mechanosensory habituation with food [33].
	Creates a negative feedback loop for dopamine release [52].		Does not perform area-restricted searching behaviour to look for food [32].	Necessary for appetitive associative learning of butanone and liquid food [78].
			Less able to learn bacterial avoidance under mitochondrial stress [36].	
			Habituates to a mechanosensory stimulus slower than wild-type worms when no food is present [33].	
			Reduced chemotaxis index to butanone paired with liquid food after conditioning [78].	

## The role of dopamine in learning, memory, and forgetting

Dopamine is involved in learning and memory formation in *C. elegans* and other animals. Learning and memory are important neurological processes and are often interconnected: ‘learning’ is the acquiring of new knowledge about changes that occur within the surrounding environment [12,21,54,55]. Learning can be divided into two main categories: ‘non-associative’ and ‘associative’ learning. After knowledge is obtained, it needs to be encoded and stored so that it may be retrieved and utilized by the organism later [12,21,54–56]. This process of encoding, storing, and retrieving information is referred to as ‘memory’. Learning and memory are highly interconnected, and it is often necessary to consider both when addressing one of these concepts [21,54,55]. Additionally, ‘forgetting’ is a distinct neurological process by which the brain discards information that was previously stored [56–58]. Like learning and memory, forgetting appears to be conserved across multiple organisms. The neurotransmitters involved in the process of forgetting have begun to be explored, with some studies showing that dopamine plays an important role [56,59–62].

### Dopamine in non-associative learning in *C. elegans*

Non-associative learning involves behavioural changes in response to exposure to one stimulus and is often seen as the simplest form of learning [54,63]. This type of learning can be further divided into the subcategories of habituation, where there is a decreased behavioural response to a repeated stimulus, and sensitization, where there is an increased behavioural response to stimuli after exposure to an intense or noxious stimulus [63,64].

A common behavioural paradigm in habituation studies with *C. elegans* is stimulating the worms’ mechanosensory receptors by tapping the Petri dish on which they are housed (causing vibrations), or stroking the worm across their body with a delicate tool such as an eyelash pick, triggering backward movement (a reversal) [22,47,55]. Repeated stimuli at consistent intervals cause the worms to habituate to the stimuli and reduces the magnitude of reversals as a response. Kindt et al. studied the role of dopamine in habituation in response to mechanosensory stimulation (repeated taps), and how this interacts with behavioural responses to food, which are partially regulated by dopamine. When food is present, dopamine deficient *cat-2* mutants and dopamine receptor *dop-1* mutants habituate faster in response to repeated taps than wild-type worms [22,33]. Interestingly, when food is absent, wild-type worms habituate more quickly compared with when food is present. This effect was not observed in *dop-1* mutants, i.e. *dop-1* mutant animals show similar rates of habituation when on or off food. In fact, when food is absent, there is no statistically significant difference in habituation rates between *dop-1* mutants and wild-type worms [33]. This indicates that worms maintain a robust escape response longer in the presence of food, a behaviour regulated by dopamine signalling. The authors speculate that one reason for this is that predators are more likely to be present when food (bacteria) is present, making it advantageous for the worm to remain more attentive to ‘danger’ cues in these conditions [33]. Another possibility is that internal feeding status impacts neuronal signalling mechanisms important for learning. Indeed, fed state has been shown to impact chemosensory responses and the expression of some neuronal receptors [65–68]. In addition, dopaminergic neurons are thought to be responsive to changes in internal state, including nutritional status [36,69].

In a gentle-touch assay, *dop-2* dopamine receptor mutant worms also habituate more quickly than wild-type worms, although no interaction with food presence was observed [47]. These studies showed that when anterior touch occurs, the dopaminergic CEP and ADE neurons modulate anterior mechanoreceptor ALM and AVM neurons to stimulate backwards movement, whereas posterior stimulation triggers the dopaminergic PDE neurons to modulate posterior mechanoreceptor PLM neurons to produce forward acceleration [22,33,47]. Taken together, although *dop-1* and *dop-2* play a significant role in habituation in response to repeated mechanical stimulation, only *dop-1* appears to also regulate food detection. This indicates that D1 and D2-like dopamine receptors in the worm, like in higher organisms, have both overlapping and separable functions.

Non-associative learning studies have also been performed with chemical odorants in *C. elegans*. Like with mechanosensory habituation, the *C. elegans* gustatory response is state-dependent, i.e. dependent on context such as the presence or absence of food [54]. Repeated or prolonged stimulation with the soluble repellent CuCl_2_ results in decreased behavioural responses [70]. When food is present, wild-type worms habituate more slowly when repeatedly stimulated with CuCl_2_ than when food is absent. Dopamine deficient *cat-2* mutant worms habituated more rapidly than wild-type animals in the presence of food, indicating that dopamine may be required for food-dependent slowing/inhibition of habituation to repeated CuCl_2_ exposure. Consistent with this, addition of exogenous dopamine to food-free assay plates causes wild-type worms to habituate more slowly than untreated controls, suggesting that dopamine mimics the inhibitory effect of food on the gustatory avoidance response. [71]. CuCl_2_ is sensed by the polymodal nociceptive neuron ASH. Repeated activation of ASH using optogenetic blue light stimulation also results in habituation – like in experiments with repeated CuCl_2_ exposure, *cat-2* mutant animals showed more rapid habituation to repeated optogenetic activation of ASH [72].

Dopamine has also been implicated in behaviours related to the other form of non-associative learning, sensitization, where pre-exposure to repellent odours can cause an enhanced sensory response. Repeated exposure to the repellent odour 2-nonanone led to increased avoidance behaviour, this effect was not observed in *cat-2* dopamine biosynthesis or *dop-3* dopamine receptor mutant animals. Although the authors do not consider this behaviour a form of sensitization, they concluded that it is a form of non-associative learning that requires dopamine signalling [73]. A study in mice showed that exposure to high salt concentrations activates sour and bitter taste-sensing cells, two key aversive taste pathways [74]. Although a similar study has not yet been performed in *C. elegans*, it is possible that exposure to high salt activates aversive pathways in an analogous manner. Indeed, high-salt concentrations could trigger pathways for osmotic stress in *C. elegans* [75].

Habituation in response to mechanosensory or chemosensory cues in *C. elegans* involve different sensory modalities detected by distinct sensory neurons. However, both forms of habituation require dopamine. This suggests that the commonality between habituation mechanisms requiring dopamine is downstream of sensory neurons, potentially acting in interneurons or the motor circuit. Further investigation into the precise circuit changes driven by dopamine signalling during habituation, as well interactions with other neurotransmitters/neuromodulators required for this form of learning, will provide interesting and important insight into how habituation is encoded in *C. elegans* and potentially in bigger brains.

### Dopamine in associative learning in *C. elegans*

Associative learning occurs when an association is made between two stimuli such that the innate behavioural response to one stimulus (the unconditioned stimulus) takes place when the second stimulus (the conditioned stimulus) is presented by itself [54,76]. This type of learning can be in response to a stimulus that is rewarding (appetitive) or noxious (aversive). Appetitive and aversive learning often involve distinct mechanisms [76], although dopamine has been shown to play an important role in both [47,77,78].

An example of an appetitive learning paradigm requiring dopamine involves pairing the odour butanone with the presence of food. In naïve worms, butanone is a mildly attractive odour. However, pairing the presence of butanone with the presence of food (a strongly attractive stimulus) leads to a learned increase in attraction to butanone [79,80]. In a recent study [78], this learned response is significantly impaired in worms lacking *cat-2*, which have reduced dopamine signalling, and in transgenic worms in which dopaminergic neurons are genetically ablated. This indicates that dopamine is required to learn the association of butanone with food [78]. In this study, the ‘conditioning’ step involves presenting food to worms in liquid media – given the involvement of dopamine in regulating swimming-dependent locomotion behaviours and swim-to-crawl transitions, it may be interesting to test in future if dopamine is required for olfactory associative learning when food is instead presented on solid media.

A study examining the role of a DEG/ENaC channel ASIC-1 in learning in *C. elegans* proposed a possible mechanism through which dopamine regulates appetitive olfactory conditioning [81]. This study used a behavioural paradigm pairing soluble (NaCl) and volatile (isoamyl alcohol) chemicals with food, showing that forming a learnt association these two cues not only requires dopamine but also requires ASIC-1 expression in dopaminergic neurons. The authors next asked how ASIC-1 could modulate dopamine release – they used super-ecliptic pHlourin (SEpHluorin) as a readout of synaptic vesicle exocytosis (and hence neurotransmission) and showed that neurotransmission was substantially reduced in dopaminergic neurons in *asic-1* mutants. They also showed that learning leads to increased neurotransmission in dopaminergic neurons in wild-type, and that this occurs to a lower extent in *asic-1* mutants. These data indicate that dopamine release, which is required for learning, is compromised in mutants lacking *asic-1* [81]. As ASIC-1 was recently shown to function in parallel with other mechanoreceptive ion channels in dopamine neurons – ENaC-like UNC-8/DEL-3 channels as well as TRP-4 and K2P-family potassium channel subunit TWK-2 [82] – it is possible that these channels also contribute to dopamine release during learning.

An example of dopamine involvement in an aversive learning paradigm was conducted by pairing isoamyl alcohol with the absence of food (starvation) [47]. Isoamyl alcohol is normally mildly attractive to naïve, wild-type worms. Pairing isoamyl alcohol with starvation leads to a learned aversion to this chemical. *dop-2* dopamine receptor mutants were unable to learn this association to the same extent as wild-type animals. This effect could be rescued when conditioning was performed in the presence of exogenous dopamine [47], suggesting that excess dopamine can bypass the requirement for D2-like dopamine receptors in this form of learning. In a test for aversive gustatory learning, Hukema et al. showed that exposure to high concentrations (100 mM) of NaCl in the absence of food led to worms changing their preference for a lower concentration (25 mM) of NaCl from attraction to repulsion, a phenomenon termed ‘gustatory plasticity’. Gustatory plasticity requires dopamine signalling, potentially functioning in parallel with serotonin [83].

Benzaldehyde adaptation when ethanol was present during conditioning was found to be dependent on the testing conditions, a phenomenon called ‘state-dependent learning’ [84]. Wild-type worms learned benzaldehyde adaptation after 90-min conditioning; worms that were conditioned in the presence of ethanol only displayed the behaviour if also tested with ethanol present. Dopamine-deficient *cat-2* mutant worms did not exhibit this state dependency [85]. This confirms the important of dopamine in state-dependent learning.

Dopamine is also essential to an interesting form of behavioural plasticity whereby *C. elegans* lay their eggs off the food lawn after exposure to biting predators *Pristionchus uniformis* (*P. uniformis*), and maintain this behaviour for several hours [86]. Both *cat-1* mutants and *cat-2* mutants have a decreased probability of laying eggs off food when conditioned with *P. uniformis*. Testing dopamine receptor mutants individually and together showed that dopamine acts through a combination of both D1- and D2-like dopamine receptors for this behaviour [86].

Like for non-associative learning, the role of dopamine in associative learning is not limited to a singular sensory modality, and is also not specific to appetitive or aversive learning [47,80]. Using the experimentally tractable brain of *C. elegans* to uncover the specific cells and circuits required for each of these sub-categories of learning, in response to distinct sensory modalities, will reveal how neuromodulators like dopamine can broadly regulate behavioural plasticity in a range of learning paradigms.

### Dopamine in the process of memory formation in *C. elegans*

Memory is divided into two main categories: short-term and long-term memory. The terms ‘long-term’ vs. ‘short-term’ refers to the process of encoding the memory and its vulnerability to decay [87–89]. In *C. elegans*, ‘short-term’ refers to memory that is assayed for 1-2 hours after learning, whereas ‘long-term’ usually refers to memory tests 12–24 hours after learning [76]. Dopamine has been shown to modulate short-term associative memory in *C. elegans*: dopamine-deficient *cat-2* mutant worms retained the memory of a learnt association between the odour butanone and the presence of food for a shorter duration compared with wild-type animals [78]. Moreover, the addition of exogenous dopamine during the conditioning step (when butanone and food are paired) resulted in longer retention of the memory of this learnt association [78]. This suggests that dopamine signalling is required during memory formation, and is also involved in retaining this memory over time. Although dopamine has been implicated in long-term memory in several mammalian models [90,91], this has not been directly tested in *C. elegans*.

### Forgetting in *C. elegans* and *D. melanogaster*

Forgetting is a neural process in which previously encoded memories cannot be retrieved. The two classical models of forgetting – ‘decay’ and ‘interference’ – suggest that forgetting is either a passive decay of the memory, or an active process in which memory traces compete for ‘space’ in the brain, respectively [57,92]. Many recent studies, discussed below, have suggested that forgetting is more likely to be an active process mediated by molecules acting in neurotransmission, second messenger signalling, and cytoskeletal modifications.

Behavioural tests for forgetting normally involve assaying for the learnt behaviour at different time points after learning/conditioning has taken place, and consequently observing when the magnitude of the learnt behavioural response returns to the level of naïve/unconditioned animals. Forgetting studies in *C. elegans* have indicated that it is an active process that requires protein synthesis [12,61]. Although the role of dopamine in forgetting has not been extensively tested in *C. elegans*, its involvement in modulating the retention of short-term associative memory as discussed above [78] indicates that it is likely to also modulate forgetting. Other neurotransmitters that have been shown to regulate forgetting in *C. elegans* include serotonin [61] and acetylcholine [62].

In *C. elegans*, two major regulatory mechanisms have been implicated in active forgetting, both of which were identified in behavioural paradigms testing olfactory responses to the attractive odorant diacetyl. ‘Olfactory adaptation’ is where pre-exposure to an odour results in weaker chemoattraction to the same odour upon subsequent exposure [58]. Genetic screens for longer retention of olfactory adaptation to diacetyl revealed the involvement of the highly conserved TIR-1 (Toll/interleukin-1 resistance domain protein) protein that acts through the MAP kinase pathway, specifically JNK, to modulate neurosecretion [58]. Follow-up research implicated multiple additional players in TIR-1/JNK-1 mediated forgetting of olfactory adaptation, including the membrane protein MACO-1, and receptor tyrosine kinase SCD-2 (and its putative ligand, HEN-1) [93], as well as diaceylglycerol (DAG) kinase DGK-1, which modulates the levels of the lipid second messenger DAG [94]. In ‘olfactory conditioning’, diacetyl is paired with starvation during a conditioning step, resulting in a learnt association between the two cues that manifests as reduced chemoattraction towards diacetyl. Using this behavioural paradigm, the RNA binding protein MSI-1/Musashi was shown to regulate forgetting by downregulating the levels of the Arp2/3 actin branching regulator complex upon associative learning [57]. Another actin regulatory protein implicated in memory loss is ADD-1/alpha-adducin [95], which acts potentially through capping actin filaments. Interestingly, although Rho family small G proteins (e.g. Rac1, Cdc42) are required for forgetting through modulating the actin cytoskeleton [96,97], the *C. elegans* Rho family member RAC-2 also regulates forgetting, but independently of actin dynamics [98].

The role of dopamine in forgetting has been more extensively explored in the vinegar fly *Drosophila melanogaster*. Multiple studies used aversive olfactory learning to assay for memory retention and forgetting in *D. melanogaster*. In this paradigm, flies are conditioned with two different odorants, the first paired with an electrical shock (conditioned stimulus +, CS+) but not the second (CS-). The flies were then allowed to choose between the two odours in a T-maze, which provides a quantifiable readout of whether the flies have learnt the association between the odour and the shock [59,60,99]. Blocking synaptic output from dopaminergic neurons after learning enhanced the memory retention of the learned pairing after three hours, whereas stimulating dopaminergic neurons after learning decreased or eliminated memory retention [99]. Interestingly, the authors found that in the mushroom body (MB) of the fly brain, a key brain region for olfactory learning and memory [100], two distinct dopamine receptors regulate memory formation and forgetting. The dDA1 dopamine receptor is essential for memory acquisition while the DAMB receptor is required for forgetting. The authors hypothesize that although these dopamine receptors are co-expressed in the same brain region, their different functions are due to the timing and context of the dopamine signal, as well as potential differences in co-transmission, second messenger signalling, and subcellular localization [60,99,101,102]. The dopamine signal required for forgetting is released by dopaminergic neurons that innervate the MB, called the MV1 neurons – interestingly, this dopamine release is correlated with behavioural state, increasing during arousal (leading to accelerated forgetting) and decreasing during rest (enhancing memory retention) [59].

The studies discussed above examine permanent forgetting, where the memory is no longer retrievable after forgetting. In contrast, transient forgetting is where the memory is only temporarily irretrievable. Transient forgetting in *Drosophila* also requires dopamine; to study this phenomenon, an ‘interrupting stimulus’ (blue light, air puff, electric shock) was presented after learning, this was shown to temporarily block the learnt behavioural response [56]. This temporary suppression of memory required a specific pair of dopaminergic neurons, PPL1-α2α’2, which innervate the MB. The dopamine signal for transient forgetting did not disrupt the cellular trace associated with memory in the MB, suggesting that transient forgetting is caused not by suppressing or replacing the existing memory, but by putting in place a temporary block on retrieval [56].

Taken together, these studies of forgetting in *Drosophila* demonstrate important roles for dopamine in memory storage, and retrieval. The well-characterized neuroanatomy of the fly model enables the dissection of specific dopaminergic cells and circuits that regulate individual aspects of learning and memory. *C. elegans*, which has a more compact and tractable nervous system that is amenable to single neuron studies, whole-brain imaging, and optogenetics, is also an excellent system to delineate the exact cells and circuits through which dopamine exerts its diverse functions. In the next section, we will discuss how advanced imaging studies, combined with the genetic accessibility of the worm, can be used to further expand our understanding of how the plasticity of neural circuits is regulated by neurotransmitter signalling.

## Visualizing dopamine signalling in neurons

Research on the role of dopamine in learning and memory currently relies heavily on genetic techniques such as knock-down or null mutations of genes involved in dopamine signalling. These are used in combination with techniques for monitoring changes in transcript/protein levels, recording neural activity via calcium signalling, and quantifying behavioural changes. Although these methods have provided important evidence implicating dopamine in regulating memory in *C. elegans*, they do not have the capacity to directly visualize dopaminergic signalling in the brain during learning, memory consolidation or retrieval. Here we describe recently-developed fluorescent biosensors for dopamine signalling, and propose their use in combination with the extensive technological toolbox in *C. elegans* to facilitate further research into the neural circuits required for learning and memory.

**Figure 3:**
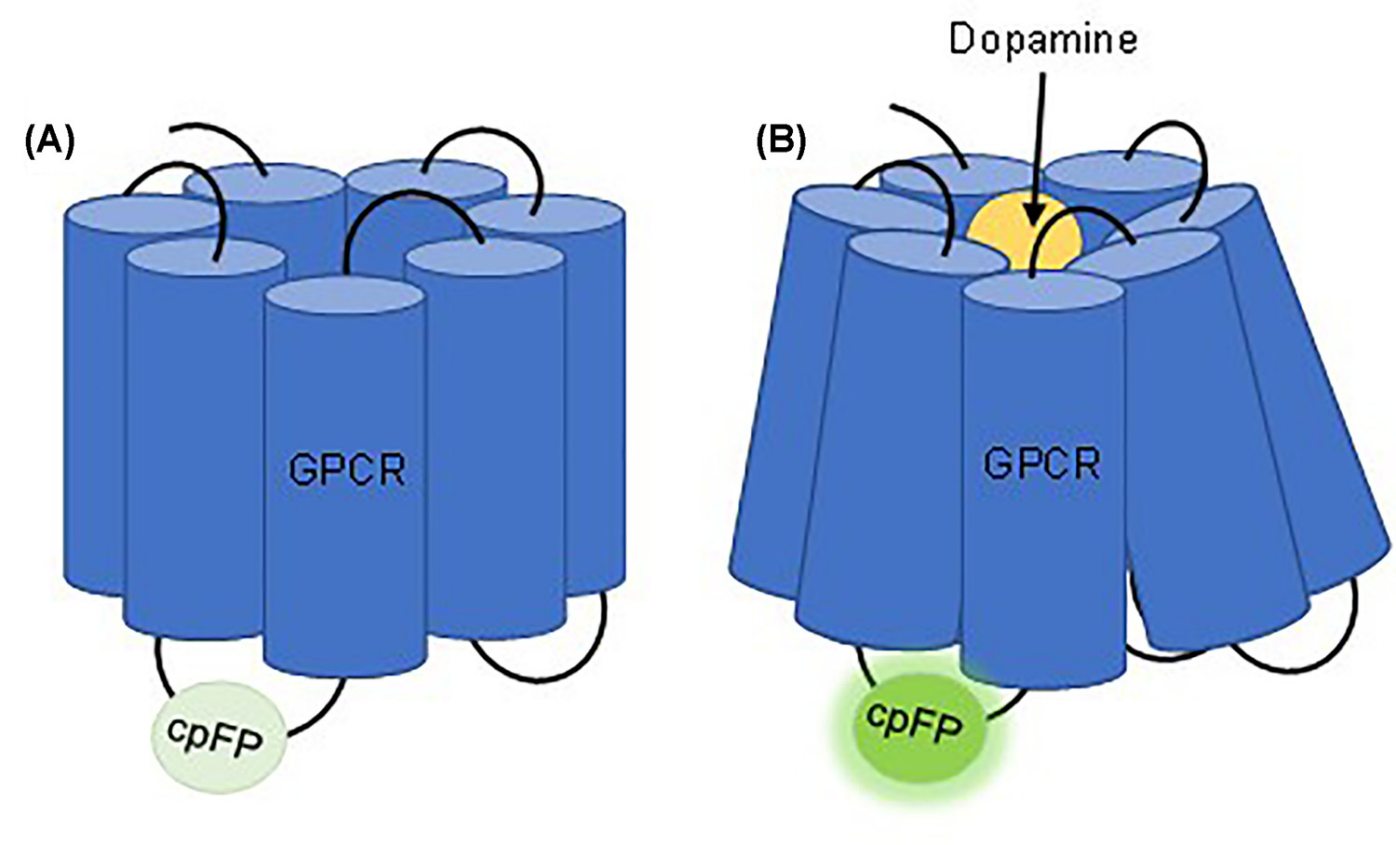
Dopamine GRAB sensor Panel (**A**) shows the GPCR with added circular permuted fluorescent protein (cpFP) on the third intracellular loop. Panel (**B**) shows the GPCR bound to dopamine, causing a conformational change in the GPCR proteins. As a result, there is a fluorescent change in the cpFP [49,103,104].

Fluorescent biosensors that detect neurotransmitter binding generally exploit structural changes to neurotransmitter-specific GPCRs upon ligand binding [49]. A fluorescent protein is inserted between the fifth and sixth transmembrane domains, where the largest conformational change occurs rapidly when the neurotransmitter binds to the receptor [103]. This conformational change causes a fluorescence change that can be measured. The fluorescent sensor can be fluorescence resonance energy transfer (FRET)-based, where two fluorescent proteins come in closer proximity during the conformational change in the GPCR, triggering a change in fluorescence [104]. The sensor can also be based on a single protein in which the conformational change in the GPCR causes changes in fluorescence intensity in the single protein. These single fluorescent protein-based sensors tend to have a better signal-to-noise ratio than the FRET-based sensors [49,105,106], and are referred to as GPCR activation-based (GRAB) sensors. In these sensors, the conformation-sensitive circular permuted fluorescent protein (cpFP) is inserted into the third intracellular loop (between the fifth and sixth transmembrane domains) (Figure 3).

Several dopamine-specific GRAB sensors have been developed, aiming for large changes in fluorescence in response to dopamine binding (dynamic range), but also sensitivity (responsive at low concentrations) and specificity (not responsive to other neurotransmitters). One example is GRAB_DA_, based on the D2 dopamine receptor, which as of now is in its second generation of development and uses two different cpFPs of different wavelengths [107,108]. The first generation of GRAB_DA_ was developed in HEK293T cells with an enhanced green version of a conformation-sensitive cpFP and human D2 dopamine receptor [107]. There were three resulting GRAB sensors, one with a high affinity to binding dopamine (DA1h), one with a medium affinity (DA1m), and a control version with mutations in the binding pocket that prevent dopamine binding (DAmut). The first generation of this sensor was reported to have high sensitivity and specificity to dopamine, however, there was some evidence of off-target effects from norepinephrine binding [107]. The second-generation GRAB_DA_ was developed to further optimize these sensors [108]. In parallel, a red fluorescent version of GRAB_DA_ was developed to facilitate simultaneous imaging with commonly used GCaMP calcium indicators, which have green fluorescence. Similar to the first-generation sensors, there were three resulting sensors for both the second generation sensors and red fluorescent sensors (h, m and mut) that differed in binding affinity. When tested in HEK293T cells, GRAB_DA_-2h had an EC50 value for dopamine of 7 nM, while GRAB_DA_-2m had an EC50 value of ∼90 nM. They were also 10- to 15-fold more selective for dopamine over norepinephrine [108].

Another dopamine-specific GRAB sensor is dLight1 [109], which unlike GRAB_DA_, is based on the D1 dopamine receptor. Two versions of dLight1 – dLight1.1 and dLight1.2 – had EC50 values for dopamine in HEK293T cells in the nanomolar range, 330 and 770 nM, respectively. These sensors were also highly selective for dopamine, the affinity for norepinephrine was 70x less than that of dopamine, the affinity for epinephrine was 40× less, and the affinity for all other neurotransmitters was negligible [109]. Like GRAB_DA_, dLight1 sensors were also generated with other wavelength options. Yellow-shifted dLight1 (YdLight1.1) and red-shifted dLight1 (RdLight1) were generated so that the sensors could be used in conjunction with other imaging tools [110]. Both GRAB_DA_ and dLight1 sensors showed good dynamic range upon dopamine binding, with 2- to 3-fold changes in fluorescence when exposed to 100 µM dopamine in HEK293T cells [108,109]. Earlier-developed tools for real-time detection of dopamine signals, such as Tango GPCR assays that rely on the detection of β-arrestin recruitment to GPCRs by ligand binding followed by measurements of downstream gene expression, generally show high sensitivity, but slower kinetics compared with GRAB_DA_ or dLight1 sensors (minutes vs seconds/milliseconds) [111].

The choice between the two different types of sensors will depend on experimental context [49,108,109,112,113]. GRAB_DA_ sensors have a higher binding affinity than the dLight1 sensors; however, dLight1 sensors have faster on- and off-rate kinetics. These parameters will need to be considered in experiments where dopamine concentrations may be low, or rapid fluctuations in dopamine levels are occurring. GRAB_DA_ sensors have a greater affinity for the norepinephrine, which is structurally similar to dopamine, than dLight1 sensors. For experiments where norepinephrine binding could be a confounding factor, dLight1 sensors may be more beneficial.

Both GRAB_DA_ and dLight1 sensors have been validated in mouse brain slices and in freely moving rodent studies, with GRAB_DA_ sensors also tested in *D. melanogaster* [49,107–110]. These *in vivo* experiments confirmed that fluorescence signals from both sensors were dependent on dopamine binding, as treating slices/animals with a D1 receptor antagonist (for dLight1) or D2 receptor antagonist (for GRAB_DA_) blocked this signal. In addition, red-shifted versions of both sensors could be experimentally combined with GCaMP6 in animal studies. This enables simultaneous calcium imaging with measurements of local dopamine transients, allowing spatiotemporal changes in dopamine signalling to be correlated with changes in neural activity [108,110]. Both GRAB_DA_ and dLight1 could also be used *in vivo* alongside optogenetics tools to activate neurotransmitter release from dopaminergic neurons and subsequently detect the response to dopamine in relevant brain regions [107–110].

Although these sensors have not been validated for use in *C. elegans*, they could be a beneficial tool in worm studies examining the role of dopamine in learning and memory. *C. elegans* are genetically tractable and have a more compact nervous system than mice or flies, making them more amenable to cell- or circuit-specific transgenic expression of GRAB sensors. Importantly, the worm is transparent, meaning that fluorescence does not need to be measured by invasive probes inserted into the brain but can rather be imaged directly under a microscope. In addition, *C. elegans* optogenetics experiments are more technically straightforward as they can be performed by illuminating worms with a bright light of the appropriate wavelength rather than inserting optical fibres into the brain. Optogenetic approaches could be used to activate the small number of dopaminergic neurons in the worm, followed by directly visualizing the spatial and temporal dynamics of resulting dopamine transients in cells of interest to identify circuit changes associated with memory – this is much more feasible in *C. elegans* than in organisms with more complex nervous systems. For example, Tanimoto *et al*. developed a new tool for behavioural tracking combined with optogenetics that enabled activation of single neurons, allowing them to identify distinct roles for individual dopaminergic neurons (CEPV vs CEPD vs PDE) in locomotion responses to food [114]. Another study used a sophisticated imaging method to record *C. elegans* movements over several hours, extracting information relating to locomotion, egg-laying, pharyngeal pumping and other motor programs. They used this method to show that dopamine is required to couple egg-laying with the roaming state [115]. Using such methods in combination with studies of learning could reveal detailed mechanistic insights into the requirement of dopamine and other neurotransmitters. Finally, new tools developed for whole brain imaging in *C. elegans*, such as the NeuroPAL tool that uses multicolour imaging to disambiguate all neurons in the worm, can be combined with GRAB sensors to observe how dopamine interactions take place in real time in an entire nervous system [116,117]. These approaches are particularly useful when studying behavioural plasticity, which involves rapid neurotransmitter-driven changes in neural circuits upon learning, including changes in the timing of neural responses [99] or the recruitment of different neurons to the circuit [118]. Taken together, using GRAB sensors in *C. elegans* together with existing imaging tools could give us a better understanding of dopaminergic neural circuits at a single neuron resolution, allowing for further insight into the role of dopamine at a cellular level that is not achievable in other experimental brains.

## Concluding remarks

Dopamine contributes to regulating plasticity across many species, including humans. In this review, we have detailed the role that dopamine plays in learning, memory, and forgetting, focusing on studies in *C. elegans*. We have also summarized the utility of recently developed fluorescent biosensors specific for dopamine, and proposed several ways they could be used to probe the spatiotemporal dynamics of dopamine signalling in the worm, at a single neuron resolution. Many diseases and disorders in humans where dopamine is implicated involve aspects of learning and memory. The highly conserved nature of dopamine signalling indicates that insights gained on how dopamine functions in learning and memory in the worm are likely to be relevant to higher organisms. Although *C. elegans* is an invertebrate animal with a compact nervous system, it displays complex behavioural plasticity that is regulated by interactions between multiple neurotransmitters and neuromodulators. Studying these processes in *C. elegans* is advantageous as it is a highly accessible experimental system that is amenable to manipulation at the molecular-, cellular- and circuit-level, enabling the detailed interrogation of how dopamine drives learning, memory, and forgetting in the entire nervous system, at high resolution. Future research that takes advantage of new imaging tools to probe memory traces and circuits in the entire nervous system of *C. elegans* is likely to provide critical insights in the context of learning and memory disorders in higher organisms, including humans.

## Data Availability

Not applicable to this review article

## Open Access

Open access for this article was enabled by the participation of Flinders University in an all-inclusive *Read & Publish* agreement with Portland Press and the Biochemical Society under a transformative agreement with CAUL.

## Abbreviations

ADE: anterior deirids
CaMKII: calcium/calmodulin-dependent protein kinase II
CEP: cephalic sensilla
cpFP: circular-permuted fluorescent protein
CREB: cAMP-response element-binding protein
DAG: diaceylglycerol
FRET: fluorescence resonance energy transfer
GPCR: G protein-coupled receptor
GRAB: GPCR activation-based
L-DOPA: L-dihydroxy-phenylalanine
PDE: posterior deirids
